# Adenosine Receptor Heteromers and their Integrative Role in Striatal Function

**DOI:** 10.1100/tsw.2007.211

**Published:** 2007-11-02

**Authors:** Sergi Ferré, Francisco Ciruela, César Quiroz, Rafael Luján, Patrizia Popoli, Rodrigo A. Cunha, Luigi F. Agnati, Kjell Fuxe, Amina S. Woods, Carme Lluis, Rafael Franco

**Affiliations:** ^1^ National Institute on Drug Abuse, Intramural Research Program, National Institutes of Health, Department of Health and Human Services, Baltimore, MD 21224, USA; ^2^ Department of Biochemistry and Molecular Biology, Faculty of Biology, University of Barcelona, 08028 Barcelona, Spain; ^3^ Department of Medical Sciences, Faculty of Medicine, University of Castilla-La Mancha, 02006 Albacete, Spain; ^4^ Department of Drug Research and Evaluation, Istituto Superiore di Sanita, 00161 Rome, Italy; ^5^ Center for Neuroscience of Coimbra, Faculty of Medicine, University of Coimbra, 3004-504 Coimbra, Portugal; ^6^ Department of Biomedical Sciences, University of Modena and Reggio Emilia, 4100 Modena, Italy; ^7^ Department of Neuroscience, Karolinska Institute, 17177 Stockholm, Sweden

**Keywords:** receptor heteromers, adenosine receptors, dopamine receptors, metabotropic, glutamate receptors, local module, striatum

## Abstract

By analyzing the functional role of adenosine receptor heteromers, we review a series of new concepts that should modify our classical views of neurotransmission in the central nervous system (CNS). Neurotransmitter receptors cannot be considered as single functional units anymore. Heteromerization of neurotransmitter receptors confers functional entities that possess different biochemical characteristics with respect to the individual components of the heteromer. Some of these characteristics can be used as a “biochemical fingerprint” to identify neurotransmitter receptor heteromers in the CNS. This is exemplified by changes in binding characteristics that are dependent on coactivation of the receptor units of different adenosine receptor heteromers. Neurotransmitter receptor heteromers can act as “processors” of computations that modulate cell signaling, sometimes critically involved in the control of pre- and postsynaptic neurotransmission. For instance, the adenosine A_1_-A_2A_ receptor heteromer acts as a concentration-dependent switch that controls striatal glutamatergic neurotransmission. Neurotransmitter receptor heteromers play a particularly important integrative role in the “local module” (the minimal portion of one or more neurons and/or one or more glial cells that operates as an independent integrative unit), where they act as processors mediating computations that convey information from diverse volume-transmitted signals. For instance, the adenosine A_2A_-dopamine D_2_ receptor heteromers work as integrators of two different neurotransmitters in the striatal spine module.

## ADENOSINE RECEPTOR HETEROMERS AS PROCESSORS OF COMPUTATIONS THAT MODULATE CELL SIGNALING

In the present review, we adopt the broad definition of "neurotransmitter" by Snyder and Ferris[1], i.e., a molecule, released by neurons or glia, which physiologically influences the electrochemical state of adjacent cells. This definition allows the inclusion of the term "neuromodulator", often used to describe adenosine. Adenosine plays a very important modulatory role in many brain processes and in brain function in general. In the brain, adenosine acts mainly by stimulating two subtypes of G protein-coupled receptors (GPCRs): adenosine $A_1$ and $A_{2A}$ receptors[2]. It is becoming evident that the modulatory role of adenosine involves the ability of adenosine receptors to heteromerize with many different partners, such as dopamine and glutamate receptors (see below). Neurotransmitter receptor heteromers are functional entities with distinctive biochemical properties different from those of the individual components of the heteromer. These biochemical characteristics include changes in ligand binding characteristics and signaling[3,4,5,6]. A receptor unit in the heteromer can display several biochemical properties, which can be simply dependent on the presence of the other unit, i.e., just as a consequence of the heteromerization, or on costimulation of the two (or more) receptor units in the heteromer. In case of dependence on costimulation, the neurotransmitter receptor heteromer acts as a "processor" of computations that modulate cell signaling. Thus, the quantitative or qualitative aspects of the signaling generated by stimulation of either receptor unit in the heteromer are different from those obtained during coactivation. This implies a processing of information at the membrane level of the signals impinging on the heteromer. Importantly, the biochemical characteristics of the receptor heteromer, which can be demonstrated in an artificial cell system, can constitute a "biochemical fingerprint" that allows its identification in the central nervous system.

The changes in binding characteristics that are dependent on coactivation of the receptor units of the receptor heteromer are a common property of neurotransmitter receptor heteromers and are also known as "intramembrane receptor-receptor interactions"[5]. The term "intramembrane receptor-receptor interaction" implies an intermolecular cross-talk between both receptor units in the heteromer at the membrane level, without intervention of signaling pathways[5]. In some cases, stimulation of one receptor unit decreases, while in other cases increases, the affinity of the other receptor unit for endogenous or exogenous ligand binding. Intramembrane receptor-receptor interactions can be unidirectional or reciprocal, with only one receptor unit or both receptor units of the heteromer being able to modulate the binding characteristics of the other receptor unit, respectively. In the $A_{2A}$-$A_1$ and the $A_{2A}$-dopamine $D_2$ receptor heteromers, there is a unidirectional antagonistic intramembrane interaction. Thus, stimulation of $A_{2A}$ receptors decreases the affinity of the other receptor units in the heteromer ($A_1$ and $D_2$ receptors) for their respective agonists (see below). The $A_1$-$A_{2A}$ receptor heteromer processes information carried by the same neurotransmitter (see below). On the other hand, the $A_{2A}$-$D_2$ receptor heteromer integrates signals from two different neurotransmitter systems, allowing adenosine to control the effects of dopaminergic neurotransmission antagonistically (see below).

Among the changes in signaling, changes in G protein coupling are another common characteristic of neurotransmitter receptor heteromers[3,4,5,6]. In several receptor heteromers, such as opioid or dopamine receptor heteromers[7,8], the receptor units in the heteromer couple to G proteins other than those usually associated with the individually expressed receptors. An interesting example in relation to adenosine receptor heteromers is the recently described $A_{2A}$-cannabinoid $CB_1$ receptor heteromer[9]. The $CB_1$ receptor signals through coupling to $G_i$ proteins, but in the $A_{2A}$-$CB_1$ receptor heteromer, the $CB_1$ receptor does not couple to $G_i$ protein unless there is coactivation of the $A_{2A}$ receptor[9]. These interactions in the $A_{2A}$-$CB_1$ receptor heteromer can have important implications for striatal function. Thus, it has been found that $A_{2A}$ and $CB_1$ receptors coimmunoprecipitate from extracts of rat striatum, where they colocalize in fibrillar structures (nerve terminals or dendritic processes)[9]. Recent results suggest that striatal $A_{2A}$-$CB_1$ receptor heteromers mediate the motor-depressant effects of cannabinoids[9]. Finally, receptor heteromerization creates an optimal frame for a tight cross-talk between the receptor units at the level of signaling, such as the strong and selective synergistic interactions between $A_{2A}$ and the metabotropic glutamate $mGlu_5$ receptor in the $A_{2A}$-$mGlu_5$ receptor heteromer at the adenylyl-cyclase and MAPK levels (see below).

## THE ADENOSINE $A_1$-$A_{2A}$ RECEPTOR HETEROMER: A CONCENTRATION-DEPENDENT SWITCH THAT CONTROLS STRIATAL GLUTAMATERGIC NEUROTRANSMISSION

In some cases, neurotransmitter receptor heteromers can act as processors of computations that modulate signaling which is critically involved in pre- or postsynaptic neurotransmission. This is exemplified by analyzing the function of the striatal $A_1$-$A_{2A}$ receptor heteromer. By means of coimmunoprecipitation and BRET techniques, we demonstrated the existence of $A_1$-$A_{2A}$ receptor heteromers in cotransfected human embryonic kidney (HEK) cells[10]. We then demonstrated the existence of an intermolecular cross-talk, an intramembrane receptor-receptor interaction, in the $A_1$-$A_{2A}$ receptor heteromer in cotransfected HEK cells, by means of radioligand binding techniques[10]. In cells only transfected with $A_1$ receptors, competitive-inhibition experiments with the radiolabeled $A_1$ receptor agonist $[^3H]$R-PIA and the $A_{2A}$ receptor agonist CGS 21680 showed that CGS 21680 displaces $A_1$ receptor binding only at high concentrations, when it loses its selectivity for $A_{2A}$ receptors. On the other hand, in cells cotransfected with $A_1$ and $A_{2A}$ receptors (but not in mixtures of cells cotransfected with either $A_1$ or $A_{2A}$ receptors), low concentrations of CGS 21680 also counteract $A_1$ receptor binding. This shows the existence of an intramembrane interaction in the $A_1$-$A_{2A}$ heteromer, by which stimulation of $A_{2A}$ receptor decreases the affinity of $A_1$ receptor for agonist binding. We could then use this biochemical characteristic of the heteromer as a biochemical fingerprint and identify the $A_1$-$A_{2A}$ receptor heteromer in the brain. In fact, the same results were obtained when we performed the same kind of competitive inhibition experiments in membrane preparations from rat striatum[10]. This demonstrates the existence of $A_1$-$A_{2A}$ receptor heteromers in the striatum. It also shows that an important part of the $A_1$ receptors in the striatum are forming heteromers with $A_{2A}$ receptors, otherwise the intramembrane $A_1$-$A_{2A}$ receptor interaction would not be detected.

We then wanted to know about the localization and functional relevance of the intramembrane interaction in the $A_1$-$A_{2A}$ receptor heteromer. In previous studies using the *in vivo* microdialysis technique in freely moving rats, we found that either perfusion with the $A_{2A}$ receptor agonist CGS 21680 or the $A_1$ receptor antagonist CPT in the ventral striatum (in the shell of the nucleus accumbens) induced a dose-dependent increase in glutamate release. The effect was counteracted by an $A_{2A}$ receptor antagonist (MSX-3) in both cases[11]. These results suggested that in the striatum, $A_1$ and $A_{2A}$ receptors could be colocalized in glutamatergic terminals, where they would exert opposite effects on the modulation of glutamate release. This was confirmed by electron microscopy experiments, labeling $A_1$ receptors with immunoperoxidase and $A_{2A}$ receptors with immunogold. Interestingly, presynaptic $A_1$ and $A_{2A}$ receptors were mostly found inside the synapse[10]. Thus, they are in a position to modulate adenosine generated by synaptically released ATP, which is most probably coreleased with glutamate and converted to adenosine by ectonucleotidases (see below). Furthermore, immunocytochemical experiments in striatal nerve terminal preparations showed that the majority of glutamatergic nerve terminals contain both $A_1$ and $A_{2A}$ receptors[10].

So, how does this heteromer work? Why do we have two receptor subtypes of the same neurotransmitter so closely interacting in the glutamatergic terminals? In preparations of striatal nerve terminals, stimulation of the $A_1$ receptor with the $A_1$ receptor agonist CPA, decreases potassium-induced glutamate release and stimulation of $A_{2A}$ receptors with CGS 21680 potentiates glutamate release[10]. Importantly, when both $A_1$ and $A_{2A}$ receptors are stimulated, there is also potentiation of glutamate release and, in the same kind of preparation, low concentrations of adenosine inhibit, while high concentrations stimulate, glutamate release[10]. In fact, previous *in vitro* experiments indicated a higher affinity for adenosine of the $A_1$ compared to the $A_{2A}$ receptor[12]. With weak adenosine release, adenosine preferentially stimulates $A_1$ receptors. This preferential stimulation in the $A_1$-$A_{2A}$ receptor heteromer inhibits glutamatergic neurotransmission. Under conditions of stronger adenosine release, $A_{2A}$ receptor activation in the $A_1$-$A_{2A}$ receptor heteromer blocks $A_1$ receptor-mediated function, and the overall result is a facilitation of the evoked release of glutamate. Thus, the $A_1$-$A_{2A}$ receptor heteromer provides a “concentration-dependent switch” mechanism by which low and high concentrations of synaptic adenosine produce the opposite effects.

The $A_1$-$A_{2A}$ receptor heteromer gives a rationale for the existence of heteromers of isoreceptors (receptors for the same neurotransmitter) and demonstrates that neurotransmitter heteromers composed of isoreceptors with different affinities for their endogenous neurotransmitter and different signaling pathways can act as concentration-dependent processors that exert a fine-tune modulation of neurotransmission. In this case, we have a neurotransmitter released or formed in the synaptic space that acts on synaptically or perisynaptically located heteromers. A weak input results in the stimulation of the receptor with the highest affinity for the neurotransmitter, while a strong input results in the additional stimulation of the other receptor, with the establishment of the intermolecular cross-talk between both receptors an a different neuronal response (Fig. 1).

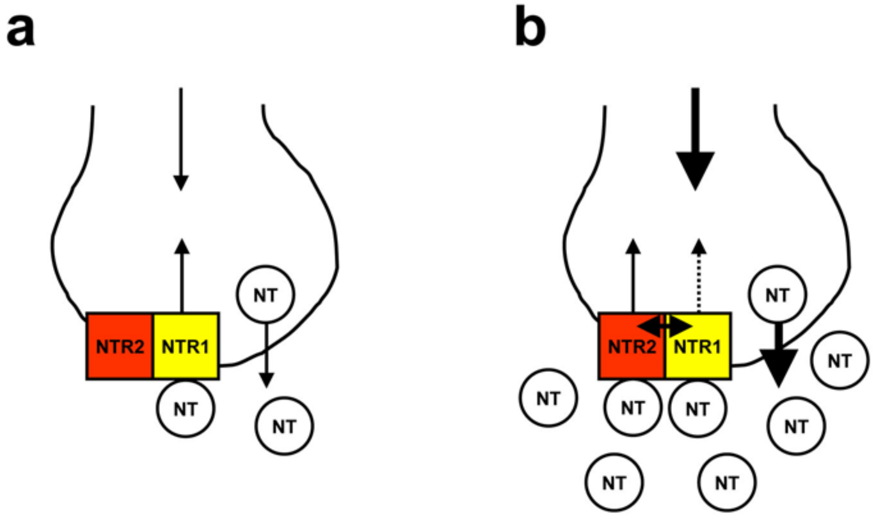

**FIGURE 1.** Heteromers of isoreceptors: “concentration-dependent switches”. (a) A weak input induces a weak neurotransmitter (NT) release, which activates the receptors with higher affinity for the neurotransmitter (NTR1). (b) A stronger input induces a stronger neurotransmitter release, with additional activation of the receptors with lower affinity (NTR2), which establishes an intermolecular cross-talk between both receptors and induces a different signaling.

In the case of heteromers of receptors for different neurotransmitters, it is still possible that they are localized in the synapse or in the perisynaptic space, where they might be activated by different coreleased neurotransmitters (Fig. 2a). The $A_{2A}$-$mGlu_5$ receptor heteromer constitutes an example, since both receptors are mainly localized in the perisynaptic space, adjacent to the postsynaptic density of the glutamatergic synapse of the GABAergic enkephalinergic neuron[13]. We have also found evidence for the existence of functional interactions between $A_{2A}$ and $mGlu_5$ receptors colocalized in striatal glutamatergic terminals[14]. The ability of $A_{2A}$ and $mGlu_5$ receptors to heteromerize was shown in transfected mammalian cells and the existence of $A_{2A}$-$mGlu_5$ receptor heteromers in the striatum was supported by coimmunoprecipitation experiments[15]. But these kinds of heteromers, if localized extrasynaptically, introduce the possibility of integrating signals conveyed by neurotransmitters released by different cells by volume transmission (extrasynaptic diffuse neurotransmission)[13] (Fig. 2b). The $A_{2A}$-$D_2$ receptor heteromer constitutes an example (see below).

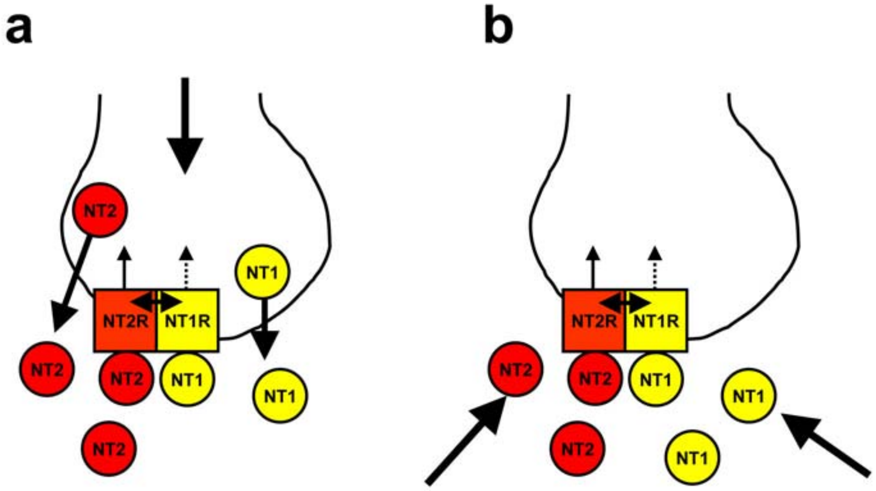

**FIGURE 2.** Heteromers of receptors for different neurotransmitters. (a) Neurotransmitter corelease. The different units of the receptor heteromer (NT1R and NT2R), which is localized in the synaptic or perisynaptic space, are activated by different neurotransmitters (NT1 and NT2) that are coreleased in the same synapse; the signaling depends on the intermolecular cross-talk. (b) Volume transmission. The different units of the receptor heteromer (NT1R and NT2R), which are localized extrasynaptically, are activated by different neurotransmitters released by different cells (NT1 and NT2) that reach the heteromer by volume transmission; the signaling depends on the intermolecular cross-talk.

## THE ADENOSINE $A_{2A}$-DOPAMINE $D_2$ AND THE ADENOSINE $A_{2A}$-GLUTAMATE MGLU$_5$ RECEPTOR HETEROMERS: INTEGRATORS OF SIGNALS IN THE STRIATAL SPINE MODULE

Altogether, their different localization (synaptic, extrasynaptic), and the different sources (neurons, glial cells) and modes (synaptic and volume transmission) of neurotransmission impinging on them, allow receptor heteromers to play a key role in the processing of computations performed by local modules[13]. The term "local module" is close to the term "local circuit" defined by Patrizia Goldman-Rakic. "Local circuit is that portion of a neuron or neurons that, under given conditions, functions as an independent integrative unit"[16]. However, this definition is too general and at the same time too restrictive. It is too general because it could include components that are involved in more than one local circuit. It is too restrictive because it does not take into account glial cells, which are now well accepted to functionally interact with neurons[17]. Furthemore, the word "circuit" implies direct "wired pathways" and extrasynaptic neurotransmission (also called "volume transmission") plays an important role at this level of computation. Thus, we have introduced the term "local module" and define it as "the minimal portion of one or more neurons and/or one or more glial cells that operates as an independent integrative unit"[13].

We will now review the role of adenosine receptor heteromers in the integration of information in the striatal spine module, the most common local module in the striatum. The GABAergic striatal efferent neuron constitutes more than 90% of the striatal neuronal population[18]. It is also called medium-sized spiny neuron, since it contains a high density of dendritic spines. The GABAergic striatal efferent neuron receives two main inputs: glutamatergic afferents from cortical, limbic, and thalamic areas, and dopaminergic afferents from the mesencephalon, either the substantia nigra pars reticulata or the ventral tegmental area, and both inputs converge in the dendritic spine[18]. The glutamatergic terminal makes synaptic contact with the head of the dendritic spine, while the dopaminergic terminal makes synaptic contact preferentially with the neck of the dendritic spine[18]. The dendritic spine, the dopaminergic and glutamatergic terminals, and astroglial processes that wrap the glutamatergic synapse constitute the most common striatal local module, which we will call striatal spine module[13].

This arrangement allows dopamine neurotransmission to regulate glutamatergic neurotransmission, but glutamate is not only released synaptically to stimulate intrasynaptic glutamatergic receptors, mostly ionotropic receptors. There is also volume transmission of glutamate, which can spill over the synaptic cleft and by an amplificatory mechanism that involves the astroglia**,** stimulates extrasynaptic receptors localized both pre- and postsynaptically at the glutamatergic and dopaminergic synapses[13]. Most of these extrasynaptic glutamatergic receptors are metabotropic glutamate receptors that modulate glutamate and dopamine release[13]. Similarly, dopamine is not only released synaptically, but can also spill over or be released by asynaptic varicosities and stimulate extrasynaptic receptors that are located both pre- and postsynaptically at both glutamatergic and dopaminergic synapses[13].

In addition to dopamine, adenosine is a very important modulator of striatal glutamatergic neurotransmission. Until recently, it was believed that the main source of extracellular adenosine was a paracrine-like formation. Extracellular adenosine would come mostly from intracellular adenosine, the concentration of which depends on the breakdown and synthesis of ATP, which is metabolized to AMP and, then, by means of 5'nucleotidases, is converted to adenosine, which can be transported to the extracelular space by means of equilibrative transporters[19]. However, recent studies suggest that astroglia plays a fundamental role in the formation of extracellular adenosine, which affects synaptic transmission. Astrocytes express glutamate (mostly metabotropic) and ATP receptors that, when activated, induce astrocytes to release glutamate and ATP[20,21]. Astroglial-released ATP can be converted to adenosine in the extracellular space by means of ectonucleotidases[22]. Finally, there is an increasing number of data that suggest the existence of a synaptic formation of adenosine, i.e., a particular synaptic pool of adenosine. In this case, adenosine would come from ATP coreleased with glutamate, which is metabolized to adenosine by means of ectonucleotidases[19]. Our finding of presynaptic $A_1$ and $A_{2A}$ receptors inside the striatal glutamatergic terminals strongly supports the functional relevance of this mechanism[10].

There are two subtypes of GABAergic striatal efferent neurons: the striatopallidal neuron, also called enkephalinergic neuron, which expresses the peptide enkephalin and dopamine and adenosine receptors of the $D_2$ and $A_{2A}$ subtypes, and the striatonigral-striatoentopeduncular neuron, also called dynorphinergic neuron, which expresses dynorphin and dopamine, and adenosine receptors of the $D_1$ and $A_1$ subtype[18,19,23]. We found the existence of antagonistic interactions between $A_{2A}$ and $D_2$ receptors that modulate the function of the enkephalinergic neuron and antagonistic interactions between $A_1$ and $D_1$ receptors that modulate the function of the dynorphinergic neuron[19,23]. We were the first to suggest that these interactions could provide a new therapeutic strategy for Parkinson's disease, mostly based on the coadministration of $A_{2A}$ receptor antagonists with L-dopa or other dopamine receptor agonists[24]. In fact, there is now clinical evidence supporting this hypothesis[25]. We and other groups demonstrated that $A_{2A}$ receptors form heteromers with $D_2$ receptors and that $A_1$ receptors form heteromers with $D_1$ receptors in transfected cells[26,27,28,29]. Importantly, we and other groups were able to demonstrate the same kind of intramembrane $A_1$-$D_1$ and $A_{2A}$-$D_2$ receptor-receptor interactions ('biochemical fingerprint") in different transfected cell lines and in the striatum[30,31,32,33,34,35,36,37,38], which demonstrates their existence in the brain.

In the $A_{2A}$-$D_2$ heteromer, the stimulation of the $A_{2A}$ receptor decreases the binding of dopamine to the $D_2$ receptor[30,31,32,33,34,35]. This intramembrane interaction controls neuronal excitability and, consequently, neuronal firing and neurotransmitter release (GABA release) by the GABAergic enkephalinergic neuron[39,40]. This is most probably related to the ability of $D_2$ receptors to suppress $Ca^{2+}$ currents through L-type VDCCs by a cAMP-PKA-independent and $G_{q/11}$-PLC-dependent signaling pathway[41]. Thus, stimulation of striatal $A_{2A}$ receptor does not produce a significant effect on its own, but it strongly counteracts the depressant effects of $D_2$ receptor stimulation on neuronal firing and neurotransmitter release[39,40]. In addition to the intramembrane interaction, a strong antagonistic interaction between $A_{2A}$ and $D_2$ receptors has been found at the second messenger level, by which stimulation of $D_2$ receptors counteracts the activation of adenylyl-cyclase induced by stimulation of $A_{2A}$

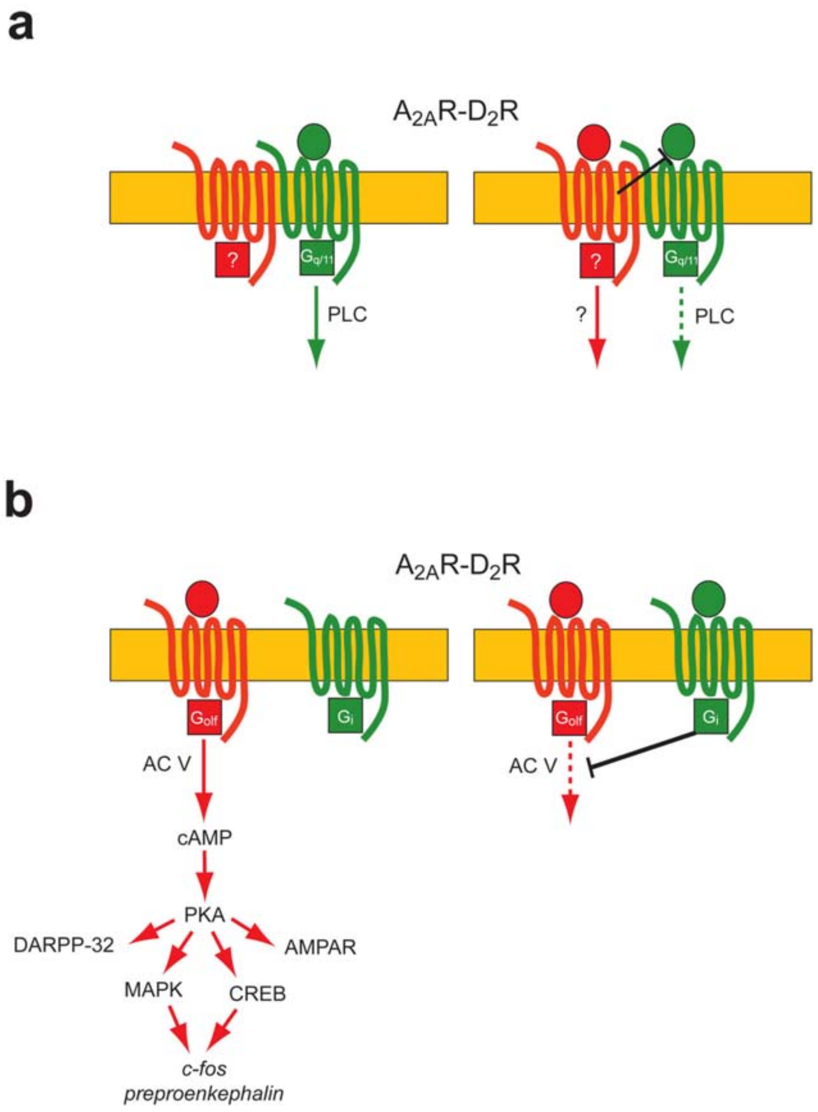

**FIGURE 3.** Adenosine $A_{2A}$-dopamine $D_2$ receptor interactions. (a) In the $A_{2A}$-D2 receptor heteromer, stimulation of the $A_{2A}$ receptor decreases the binding of dopamine to the $D_2$ receptor; this intramembrane $A_{2A}$-$D_2$ interaction seems to involve a $D_2$ receptor-$G_{q/11}$-PLC signaling pathway. (b) In addition to the intramembrane interaction, in the striatum, a strong antagonistic interaction between $A_{2A}$ and $D_2$ receptors has been found at the second messenger level, by which stimulation of $G_i$-coupled $D_2$ receptors counteracts the activation of adenylyl-cyclase (subtype V; AC V) induced by stimulation of $A_{2A}$ receptors and, therefore, the consequent activation of the cAMP-PKA-DARPP-32 signaling pathway and induction of the expression of different genes, such as *c-fos* and *preproenkephalin*; this interaction might not depend on receptor heteromerization.

receptors[27,33]. Stimulation of $A_{2A}$ receptor can potentially stimulate adenylyl-cyclase, with consequent activation of cAMP-PKA signaling pathway and induction of the expression of different genes, such as *c-fos* and *preproenkephalin*, by activating the constitutive transcription factor CREB and the MAPK pathway[19,23]. Also, $A_{2A}$ receptor-mediated activation of PKA can induce phosphorylation of DARPP-32[33] and AMPA receptors[42], which plays a crucial role in the initial plastic changes of glutamatergic synapses, which includes synaptic recruitment of AMPA receptors[43]. However, under basal conditions, stimulation of $A_{2A}$ receptors can poorly activate cAMP-PKA signaling and increase gene expression, due to a strong tonic inhibitory effect of endogenous dopamine and $D_2$ receptor stimulation on adenylyl-cyclase[13,19,42,44,45]. There is, therefore, dissociation between both $A_{2A}$-$D_2$ receptor interactions.

Either costimulation of $A_{2A}$ and $D_2$ receptors results in blockade of the $D_2$ receptor-$G_{q/11}$-PLC signaling pathway, by means of the intramembrane $A_{2A}$-$D_2$ interaction, or it results in a blockade of the $A_{2A}$ receptor-$G_{s\text{-}olf}$-cAMP-PKA signaling, by means of the $A_{2A}$-$D_2$ interaction at the adenylyl-cyclase level (Fig. 3). It is possible that when the $D_2$ receptor is not forming heteromers, it couples preferentially to $G_i$, while, when forming heteromers with the $A_{2A}$ receptor, the $D_2$ receptor couples preferentially to $G_{q/11}$. This would be a similar situation to that recently described for the $D_1$-$D_2$ receptor heteromer[8]. Nevertheless, in the $D_1$-$D_2$ receptor heteromer, both receptors couple and signal through $G_{q/11}$[8], while in the $A_{2A}$-$D_2$ receptor heteromer, the main function of the $A_{2A}$ receptor seems to be the control of $D_2$ receptor signaling through $G_{q/11}$. In this case, $A_{2A}$ receptor does not couple to $G_{s/olf}$, or else there should be activation of the cAMP-PKA signaling under basal conditions.

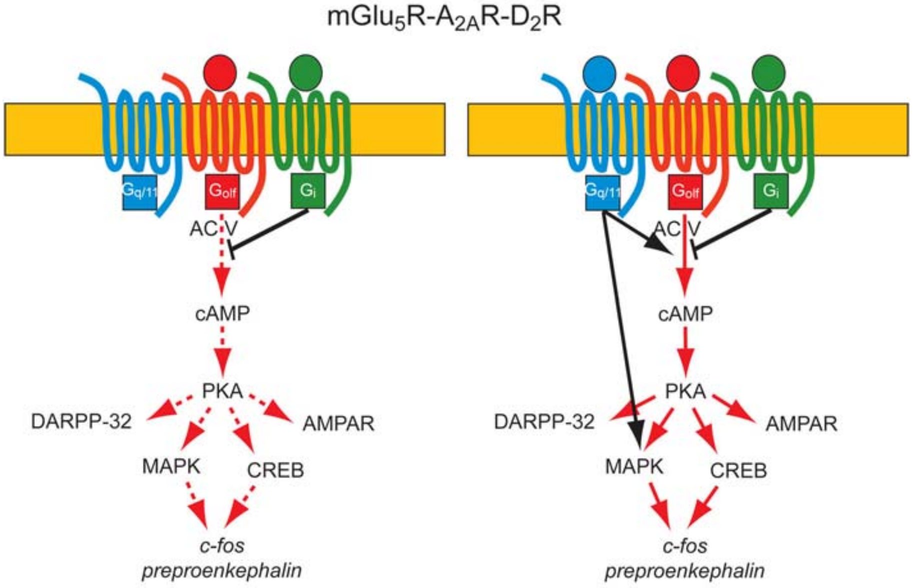

**FIGURE 4.** Metabotropic glutamate $mGlu_5$-adenosine $A_{2A}$-dopamine $D_2$ receptor heteromers. Under basal conditions, in the striatum, stimulation of $A_{2A}$ receptors can poorly activate cAMP-PKA signaling and increase gene expression, due to a strong tonic inhibitory effect of endogenous dopamine and $D_2$ receptor stimulation on adenylyl-cyclase. The $mGlu_5$ receptor heteromerizes and functionally interacts with the $A_{2A}$ receptor. By potentiating the effects of $A_{2A}$ receptor on adenylyl-cyclase and MAPK activation, $mGlu_5$ receptor coactivation allows the $A_{2A}$ receptor to counteract the inhibitory effect of $D_2$ receptor.

Which are, therefore, the conditions that allow the $A_{2A}$ receptor to activate PKA in the GABAergic enkephalinergic neuron? One possibility is to decrease dopamine $D_2$ receptor signaling at the same time that the $A_{2A}$ receptor is stimulated. We have obtained evidence suggesting that $A_1$ receptor stimulation, which is in fact achieved by release of endogenous adenosine, inhibits dopamine release and, therefore, enables $A_{2A}$ receptor costimulation to induce a selective activation of gene expression (c-fos, preproenkephalin) in the GABAergic enkephalinergic neurons[44,45,46]. Another possibility is to potentiate $A_{2A}$ receptor-mediated signaling through $G_{s\text{-}olf}$. As mentioned before, the $G_{q/11}$-coupled $mGlu_5$ receptor has been shown to physically associate with $A_{2A}$ receptors in transfected cells and in the striatum[15]. At the intramembrane level, in rat striatum, stimulation of $mGlu_5$ receptors antagonizes the binding of dopamine to the $D_2$ receptor binding, which strongly suggests that it also heteromerizes with $D_2$ receptors[47]. Furthermore, stimulation of $mGlu_5$ receptors potentiates the antagonistic effect of $A_{2A}$ receptors on $D_2$ receptor binding, suggesting the existence $mGlu_5$-$A_{2A}$-$D_2$ receptor heteromers[47]. It is, therefore, possible that in the $mGlu_5$-$A_{2A}$-$D_2$ receptor heteromers, $D_2$ receptors predominantly use $G_i$-cAMP-PKA signaling (Fig. 4). Furthermore, in transfected HEK cells, we found synergistic interactions between $mGlu_5$ receptor and $A_{2A}$ receptors, by which $mGlu_5$ receptor stimulation potentiates the effects of $A_{2A}$ receptor at the MAPK level[15]. In the same cells, stimulation of $mGlu_5$ receptor very strongly potentiated *c-fos* expression induced by $A_{2A}$ receptor stimulation, which was completely counteracted by an inhibitor of MAPK activation[15]. Previous studies have shown that activation of $G_{q/11}$-coupled receptors can amplify adenylyl-cyclase activation induced by $G_s$-coupled receptor[48]. This was not observed in transfected HEK cells[15], but in striatal slices, $mGlu_5$ receptor activation potentiates $A_{2A}$ receptor-mediated PKA activation with phosphorylation of DARPP-32[49]. Then, *in vivo*, costimulation of $mGlu_5$ receptors could allow $A_{2A}$ receptors to override the tonic inhibition imposed by $D_2$ receptors and induce an increase in gene expression (Fig. 4). In fact, we found that the central coadministration of selective $A_{2A}$ and $mGlu_5$ receptor agonists induces an increase in the striatal expression of *c-fos*, while no significant effect was obtained when they were administered alone[15].

In different behavioral models, $mGlu_5$ receptor agonists and antagonists produce similar effects to $A_{2A}$ receptor agonists and antagonists, respectively, including selective modulation of $D_2$ receptor-mediated effects. A selective $mGlu_5$ receptor agonist preferentially inhibits motor activation induced by $D_2$ receptor agonists[47], whereas $mGlu_5$ receptor antagonists counteract the effects of $D_2$ receptor antagonists[50]. Furthermore, $A_{2A}$ and $mGlu_5$ receptor agonists and $A_{2A}$ and $mGlu_5$ receptor antagonists also show synergistic effects at the behavioral level[15,47,51,52]. $A_{2A}$-$D_2$-$mGlu_5$ receptor interactions provide the rationale for the coapplication of $A_{2A}$ and $mGlu_5$ receptor antagonists in Parkinson's disease[51,52].
How do the pre- and postsynaptic heteromers that contain $A_{2A}$ receptors modulate glutamatergic neurotransmission in the striatal spines of the enkephalinergic neurons? Under weak cortico-limbic input, we have a preferential $A_1$ receptor-mediated modulation in the $A_1$-$A_{2A}$ receptor heteromer at the presynaptic side, and a preferential $D_2$ receptor-mediated modulation in the $A_{2A}$-$D_2$, and possibly $A_{2A}$-$D_2$-$mGlu_5$ heteromers, in the postsynaptic side. This provides weak glutamatergic neurotransmission, weak neuronal excitability, and weak gene expression and plastic changes. Under strong cortico-limbic input, we have a strong release of glutamate and formation of synaptic adenosine, which stimulates presynaptic $A_{2A}$ receptors in the $A_1$-$A_{2A}$ receptor heteromer, which shuts down $A_1$ receptor signaling and promotes further glutamate release. Second, synaptic glutamate and adenosine can overflow from the synaptic space and activate $A_{2A}$ and $mGlu_5$ receptors forming heteromers in the perisynaptic postsynaptic side. Thus, we have a strong activation of the $A_{2A}$ and $mGlu_5$ receptors in the $A_{2A}$-$D_2$ and possibly $A_{2A}$-$D_2$-$mGlu_5$ receptor heteromer, which shuts down $D_2$ receptor signaling and increases neuronal excitability and also allows gene expression, protein synthesis and synaptic plasticity. As mentioned above, we should also consider a possible role of $A_1$ receptor stimulation in dopaminergic nerve terminals by endogenous adenosine, which would decrease dopamine release and contribute to the decreased $D_2$ receptor signaling.

Thus, stimulation $A_{2A}$ receptors in the pre- and postsynaptic $A_{2A}$ receptor-containing heteromers seems to play a key role in the functional changes of the glutamatergic synapses of the enkephalinergic neuron during conditions of strong cortico-limbic input. In agreement, we have recently shown that $A_{2A}$ receptor blockade completely counteracts MAPK activation (phosphorylation of ERK1/2) in the GABAergic enkephalinergic neurons induced by cortical electrical stimulation[53].

This can have implications for the treatment of drug addiction: The glutamatergic projections from the prefrontal cortex to the nucleus accumbens (particularly the nucleus acumbens core) seem to play a key role in relapse to drugs of addiction[54]. Given the key role of the $A_{2A}$ receptors of pre- and postsynaptic heteromers in the glutamatergic synapses of the GABAergic enkephalinergic neurons, $A_{2A}$ receptor antagonists could provide a treatment for relapse[55].

## CONCLUSIONS

Reviewing the functional role of adenosine receptor heteromers allowed us to discover the functional relevance of neurotransmitter receptor heteromers at different levels of analysis of brain function. First, at the receptor level, a receptor unit in the heteromer can display several biochemical properties, which can be dependent on costimulation of the other unit (or units, in case of receptor heteromultimers). In this case, the neurotransmitter receptor heteromer acts as a "processor" of computations that modulate cell signaling. Second, this process of information might be involved in the modulation of cell signaling critically involved in the control of pre- and postsynaptic neurotransmission. Finally, at a higher level of analysis, neurotransmitter receptor heteromers play an important role in the computation of information performed by "local modules". This does not only depend on their intrinsic ability to process information, but on their intra- and extrasynaptic localization, which allows them to integrate signals coming from different sources (neurons, glia) and using different modes (synaptic and volume transmission) of neurotransmission. Many questions about receptor heteromers remain to be answered. For instance, we still need to determine the detailed molecular mechanisms by which heteromerization changes the biochemical characteristics of a receptor or by which stimulation of one receptor in the heteromer leads to the allosteric modification of the adjacent receptor that changes its functional characteristics. Also, in this review, we have been focusing on adenosine receptor heteromers, and adenosine does not only activate receptor heteromers, but also adenosine receptor homomers, which should obviously be taken into account when trying to fully understand the functional role of adenosine in a particular local module. In any case, the realization of the functional relevance of neurotransmitter receptor heteromers can have important implications for the treatment of neuropsychiatric disorders and drug addiction.

## ACKNOWLEDGMENTS

Work supported by the NIDA IRP funds.
